# Effect of a checklist-based core competency training and evaluation system on work-related alienation among critical care nurses

**DOI:** 10.3389/fpubh.2026.1708734

**Published:** 2026-03-18

**Authors:** Liuliu Wang, Zeyu Liu, Minghui Tong, Junyan Guo, Hongmei Zhu

**Affiliations:** Department of Cardiac Surgery Nursing, Peking University International Hospital, Beijing, China

**Keywords:** checklist management, core competency training, critical care nurses, evaluation system, work alienation

## Abstract

**Objective:**

To explore the effect of a checklist-based core competency training and evaluation system on work-related alienation among nurses in critical care settings.

**Methods:**

A quasi-experimental design was used, involving 150 critical care nurses at a single institution between June 2020 and June 2021. During the initial phase (June–December 2020), participants received conventional training and routine interventions targeting work-related alienation (pre-intervention). In the subsequent intervention phase (January–June 2021), the same nurses underwent a checklist-based core competency training and evaluation system integrated into standard practice (post-intervention). Comparisons were made between the two phases regarding training satisfaction, theoretical and practical examination scores, and work-related alienation scale scores.

**Results:**

Post-intervention data indicated significantly higher training satisfaction scores across all dimensions (*p* < 0.05). Theoretical and practical examination scores were significantly improved (both, *p* < 0.001). Furthermore, work-related alienation decreased, while perceived organizational support and person–organization fit post-intervention increased compared with pre-intervention (all *p* < 0.001).

**Conclusion:**

The implementation of a checklist-based core competency training and evaluation system was associated with improved professional competency and training satisfaction among critical care nurses. This approach contributed to enhanced perceived organizational support, better alignment between individual and organizational values, and a measurable reduction in work-related alienation. These outcomes suggest potential benefits for nursing workforce engagement and retention in high-acuity clinical settings.

## Introduction

Work-related alienation is a psychological condition characterized by a sense of detachment from work, wherein employees perceive their current roles as insufficient for meeting personal needs and expectations. This detachment may lead to adverse emotional responses and a diminished sense of purpose, ultimately impairing job performance and reducing productivity (1, 2). Within clinical nursing settings, nursing professionals represent a high-risk group for work-related alienation, attributable to factors such as inadequate staffing, excessive workloads, elevated emotional demands, and occupational hazards (3, 4). Critical care nurses are susceptible to more pronounced manifestations of work-related alienation due to the complexity of clinical tasks and the increased demands for quality and precision. This condition is associated with decreased work engagement, which may contribute to compromised patient safety, increased staff turnover, and exacerbation of existing nursing shortages (5).

Checklist-based management is a structured work management model that emphasizes standardization and strict adherence to essential clinical protocols. By implementing detailed checklists that outline specific responsibilities and procedural requirements, this approach provides targeted, systematic guidance intended to help nurses master workflows efficiently and integrate more smoothly into their professional roles (6, 7). However, existing domestic research on the structured development of core competency checklists tailored specifically to critical care nursing remains limited, particularly concerning psychological outcomes such as work alienation. To address this gap, the present study developed a checklist-based core competency training and evaluation system for critical care nurses and assessed its effectiveness through a quasi-experimental design. The study aimed to determine the impact of this system on professional competency, satisfaction with engagement, and the alleviation of work-related alienation, thereby contributing new training strategies and empirical evidence to support clinical nursing management practices.

### Theoretical framework

This study is grounded in self-determination theory, which posits that the fulfillment of three fundamental psychological needs (i.e. competence, autonomy, and relatedness) is essential for fostering intrinsic motivation and promoting psychological wellbeing. The competency-based checklist training system addresses these needs through multiple mechanisms. First, the structured checklist provides nurses with clear learning objectives and evaluation criteria, enabling them to systematically enhance their professional knowledge and skills, thereby strengthening their sense of competence. Second, the checklist-guided process of continuous self-assessment transforms nurses from passive recipients of evaluation into active participants in their own professional development, enhancing their sense of autonomy. Finally, the system emphasizes team communication and collaboration, while also reflecting organizational support, which contributes to a stronger sense of relatedness. We hypothesize that by simultaneously satisfying these three psychological needs, the training can effectively mitigate work alienation, which often stems from feelings of helplessness, lack of control, and social isolation.

## Materials and methods

### Sample size and participants

The sample size was estimated based on Kendall's rule of thumb, which recommends that the formal sample size should be 5–10 times the number of items included in the main measurement instrument. As the questionnaire contained 20 items, an appropriate sample size range was 100–200 participants. To account for potential invalid or incomplete responses, we aimed for a sample size above the lower bound of this range. Accordingly, 150 participants were recruited, which falls well within the recommended range and provides sufficient statistical robustness.

Registered nurses from critical care units including the intensive care unit and emergency department were recruited between June 2020 and June 2021, yielding a total of 150 participants. This study was approved by the hospital ethics committee of Peking university international Hospital (No. 2022-KY-0061-01), and all participants provided informed consent.

Inclusion criteria were: (1) possession of a valid nursing license with ≥ 1 year of clinical experience; (2) regularly assigned to critical care units; (3) voluntary participation with signed informed consent.

Exclusion criteria were: (1) nurses on extended leave, enrolled in external training, or rotated to non-critical care units during the study period; and (2) inability to complete the study due to other reasons.

A quasi-experimental before-after design was adopted. During the initial phase (June–December 2020) conventional and routine training were administered (pre-intervention). In the training phase (January–June 2021), a checklist-based core competency training and evaluation system was implemented for the same nurses during the post-intervention phase. The roles and professional titles of nurses remained constant throughout the study period to minimize potential confounding variables.

### Methods

During conventional training and routine interventions (pre-intervention), nurses received standard nursing training through the distribution of standardized manuals. These manuals included essential nursing theory, practical skills, and clinical competencies relevant to critical care nursing. Monthly assessments were conducted by nurse managers, who evaluated job satisfaction during individual meetings, identified manifestations of work-related alienation, analyzed contributing factors, and provided appropriate psychological support and trainings. Team cohesion activities were organized to promote a positive departmental environment and to strengthen professional relationships.

The post-intervention group received standard training along with a checklist-based core competency training and evaluation system. The development of this system involved the creation of a competency checklist for critical care nurses, guided by checklist management principles and Kirkpatrick's four-level evaluation model, and developed using the Delphi method. Initial training components were drafted based on a comprehensive literature review and analysis of institutional context. Three rounds of expert consultation were conducted with seven experts, resulting in a finalized framework comprising one first-level indicator, four second-level indicators, and 11 third-level indicators (Table 1). Specific competency items were defined under each third-level indicator, and a corresponding evaluation system was subsequently established. Building upon this framework, a corresponding evaluation system was established, detailing clear assessment standards and evaluation methods. Training and evaluation were organized under a group leader responsibility system. The overall leader supervised quality control and provided guidance to subgroup leaders. Each subgroup, comprising ward head nurses, teaching instructors, and quality control officers, was responsible for delivering training and assessment according to the specific competency checklist for each professional level.

**Table 1:** Core competency training checklist framework for critical care nurses.

**First-level indicator**	**Second-level indicator**	**Third-level indicator**	**Competency checklist content**
Basic competency	Knowledge	Familiarity with nursing policies and procedures	1. Core nursing policies and essential procedures including patient identification and verification systems, and various handover protocols. 2. Ward management standards and procedures, encompassing admission and discharge management, systems for ward operations. 3. Medication management policies, covering principles of medication administration; emergency medications, automated dispensing cabinets, refrigerated medications. 4. Guidelines for the management and prevention of common technical complications, such as oral care, nasogastric feeding, and urinary catheterization.
		Mastery of nursing-related knowledge	1. Knowledge of fundamental nursing 2. Knowledge of medical-surgical, obstetric-gynecological, and pediatric nursing
		Proper implementation of infection control knowledge	1. Standards for hand hygiene 2. Standards for aseptic technique 3. Management of disinfection and isolation 4. Occupational exposure and protection 5. Management of medical waste 6. Prevention and control of catheter-related infections
		Familiarity with critical care knowledge	1. Routine care of critically ill patients 2. Guidelines on cardiopulmonary resuscitation 3. Use and precautions of emergency medications
	Skills	Proper implementation of Nursing Skills	1) Basic life support procedures, including adult cardiopulmonary resuscitation techniques and defibrillation protocols. 2) Clinical procedural competencies, such as aseptic technique, intravenous catheter insertion, and subcutaneous injection. 3) Routine nursing procedures, including vital sign monitoring, provision of morning and evening care, indwelling urinary catheterization, and enema administration. 4) Operation and management of commonly used medical equipment, such as patient monitors, defibrillators, infusion pumps, and syringe pumps.
	Communication	Proactive effective communication	1. Communication between nurse-patient, nurse-nurse, nurse-physician 2. External communication: interdepartmental, third-party, etc.
	Practice	Active organization and coordination	1. Accurate and clear planning 2. Orderly work 3. Reasonable organization and coordination
		Timely and accurate assessment	1. Comprehensive patient assessments, including admission assessments, activities of daily living evaluations, daily clinical assessments, health education evaluations, and pain assessments. 2. Risk assessments involving evaluation of fall risk, pressure ulcer risk, nutritional risk, and risk of thromboembolism. 3. Monitoring and assessment of changes in clinical condition, including variations in symptoms and signs, atypical patient complaints, and abnormal laboratory findings (including critical values). 4. Evaluation of medical orders and nursing interventions, focusing on order standardization, appropriateness of nursing care plans, and effectiveness of implemented nursing measures. 5. Identification of potential risks, including anticipated complications, risks associated with medical and nursing practices, and environmental or equipment-related hazards.
		Resolution of nursing problems	1) Implementation of routine nursing problem identification, including assessment of issues related to admission, catheter use, diagnostic tests, and discharge processes, accompanied by the appropriate execution of nursing interventions. 2) Implementation of individualized nursing problem identification based on patient-specific conditions, addressing both current and potential nursing issues, with corresponding targeted interventions. 3) Ongoing evaluation of nursing outcomes with an emphasis on continuous quality improvement. 4) Competency in resolving additional clinical issues, including addressing individualized patient needs, responding to patient inquiries, and managing ward environment and personnel effectively.
		Implementation of health education	1. Proficiency in delivering routine health education content, including information related to admission, discharge, and medication administration. 2. Targeted implementation of health education activities, involving the structured delivery of planned content, accurate documentation of educational interventions, and timely follow-up.
		Clinical emergency capabilities for nurses	1) Recognition, assessment, management, and coordination of resuscitation efforts in response to changes in patient conditions. 2) Competent response to emergency situations, including fires, major medical events, information system failures, and suicide attempts.

The same nurses participated in pre-intervention and post-intervention.

### Outcome measures

(1) Training satisfaction was assessed using the McCloskey/Mueller Satisfaction Scale, a well-established instrument for measuring nurse job satisfaction (8, 9). It has demonstrated excellent psychometric properties, with a reported reliability coefficient of 0.89 and internal consistency (Cronbach's α) of 0.97. The version used in this study comprised three domains: effectiveness evaluation (four items), content design (three items), and willingness to participate (three items). Each item was rated on a 5-point Likert scale ranging from “very dissatisfied” (1) to “very satisfied” (5), with higher scores indicating greater satisfaction. This tool was used in its original form translated to Chinese.

(2) Examination performance was evaluated using the hospital's standardized hierarchical evaluation system, administered uniformly by the Department of Nursing. This system was developed based on the hospital's competency framework and includes both theoretical and practical assessments that reflect the core competencies required for critical care nursing. The content and difficulty of the assessments were aligned with the hospital's hierarchical training manual, ensuring consistency across both phases. The validity of the system is supported by its alignment with clinical competency standards and expert consultations. While formal statistical reliability tests (e.g., internal consistency) have not been conducted, the system has demonstrated stable and reliable results in repeated applications across different clinical units and by multiple evaluators. Inter-rater reliability is ensured through standardized training for evaluators. Scores are assigned on a 100-point scale, with higher scores reflecting greater proficiency in knowledge and skills acquisition. Data were organized using Excel spreadsheets for statistical analysis.

(3) Work-related alienation was measured before and after the training using the Work Alienation Questionnaire developed by He et al. (10). The scale consists of four dimensions (i.e. powerlessness, meaninglessness, self-alienation, and social alienation), with a total of 20 items. It adopts a five-point Likert scoring system, with an overall Cronbach's α coefficient of 0.953 and subscale reliabilities ranging from 0.814 to 0.893, indicating excellent internal consistency. This tool has been widely applied to evaluate work alienation across various occupational phases. Items were rated on a 5-point Likert scale from “completely disagree” (1) to “completely agree” (5), with higher scores reflecting a higher level of work-related alienation.

The Cronbach's α values for each scale are summarized in Supplementary Table 1.

We invited an expert team consisting of five members, including three scholars specializing in organizational behavior and human resource management (two of whom were directors of nursing departments) and two head nurses/charge team leaders with more than 10 years of management experience. The experts evaluated each item across three dimensions (i.e. relevance, clarity, and contextual appropriateness) using a 4-point rating scale (1, not relevant/unclear/inappropriate; 4, highly relevant/clear/appropriate). Item-level content validity indices were then calculated. The Cronbach's α coefficients for all items ranged from 0.943 to 0.952, indicating excellent content validity of the scale.

Ten nurses were randomly selected to participate in cognitive interviews, during which they were asked about their understanding of specific items, whether the wording was ambiguous, and their thought processes when responding. Based on their feedback, we further refined the wording and finalized a 10-item scale to improve clarity.

The example items from each instrument the McCloskey/Mueller Satisfaction Scale and the Work Alienation Questionnaire are listed below:

The McCloskey/Mueller Satisfaction Scale:

(1) Implementation of hospital nursing management systems: (i) Core nursing systems and key procedures: patient identification and verification systems, various handover procedures, etc. (ii) Ward management–related systems and processes: admission and discharge management standards, ward management regulations, etc. (iii) Medication management systems: principles of medication administration; management of emergency drugs, automated dispensing cabinets, refrigerated medications, etc. (iv) Management and prevention of common technical complications: oral care, nasogastric feeding, catheterization, and related procedures.
(2) Work control: the training program includes both basic skills training and specialized professional training, as well as training related to emergency event management. Training is conducted according to hierarchical competency requirements, with difficulty levels appropriately matched to job roles and workload intensity. As a result, nurses who have completed core competency training demonstrate greater control over their work compared with those who have not received such training.
(3) Psychological workload: the competency framework also incorporates training on clinical problem-solving and emergency response. This enables nurses to develop clearer expectations regarding their work, thereby reducing uncertainty-related psychological stress. With improved preparedness, nurses can work more efficiently and accurately, while reducing psychological burden caused by difficulties in handling work tasks. As nurses gain greater control over their professional competencies, the perceived intensity of work is also noticeably reduced.

The Work Alienation Questionnaire:

(1) Powerlessness: a significant proportion of work time is perceived to be wasted on activities that are considered meaningless.
(2) Meaninglessness: nurses may feel that their personal strengths and expertise cannot be fully utilized in their current positions.
(2) Social isolation: when problems arise at work, nurses may feel that they cannot obtain adequate support or assistance. This perception is often related to inconsistent understandings of work standards and performance expectations. When individuals are unclear about required competency levels, they may feel that their abilities are not recognized or demonstrated.

### Statistical analysis

Data were analyzed using SPSS version 26.0. Continuous variables with normal distribution were expressed as mean ± standard deviation (x ± s). Independent samples *t*-tests were applied for between-group comparisons and paired-samples *t*-tests were used for within-group comparisons. *p* < 0.05 was considered statistically significant.

## Results

### Comparison of training satisfaction

All three outcomes were significantly higher in the post-intervention phase than in the pre-intervention phase. Scores for effectiveness evaluation, content design, and participation willingness increased from 13.21 ± 1.54 to 16.39 ± 1.28, from 9.59 ± 1.53 to 12.16 ± 1.04, and from 8.59 ± 1.68 to 10.12 ± 1.31, respectively (all *p* < 0.001). These results indicate that the checklist-based core competency training was associated with significant improvements in nurses' perceptions across all assessed domains (Table 2).

**Table 2:** Training satisfaction ($\bar{x}\pm SD$, points) before and after intervention.

**Outcome**	**Post-intervention (*n* = 150)**	**Pre-intervention (*n* = 150)**	***P***
Effectiveness evaluation	16.39 ± 1.28	13.21 ± 1.54	< 0.001
Content design	12.16 ± 1.04	9.59 ± 1.53	< 0.001
Participation willingness	10.12 ± 1.31	8.59 ± 1.68	< 0.001

### Comparison of examination performance

Following the training period, significantly higher theoretical and practical examination scores were recorded in post-intervention compared with pre-intervention (*t* = 15.966, *p* < 0.001; *t* = 28.781, *p* < 0.001, respectively) (Table 3).

**Table 3:** Comparison of Examination Performance ($\bar{x}\pm S$, points).

**Group**	**Sample size**	**Theoretical examination score**	**Practical examination score**
Post-intervention	150	91.59 ± 2.57	92.14 ± 2.18
Pre-intervention	150	85.26 ± 4.12	82.49 ± 3.48
*t*	–	15.966	28.781
*P*	–	< 0.001	< 0.001

### Comparison of work-related alienation

Following the intervention, work-related alienation decreased, while perceived organizational support and person–organization fit increased significantly compared with pre-intervention levels. Specifically, work alienation scores declined from 42.14 ± 2.23 to 35.49 ± 4.59, perceived organizational support increased from 15.24 ± 2.16 to 21.19 ± 1.08, and person–organization fit improved from 11.57 ± 1.58 to 15.19 ± 1.92 (all *p* < 0.001). These results indicate that the intervention effectively reduced nurses' work alienation and enhanced their perceptions of organizational support and fit (Table 4).

**Table 4:** Work alienation, perceived organizational support, and person–organization Fit ($\bar{x}\pm SD$, points) before and after intervention.

**Outcome**	**Post-intervention (*n* = 150)**	**Pre-intervention (*n* = 150)**	***P***
Work alienation	35.49 ± 4.59	42.14 ± 2.23	< 0.001
Perceived organizational support	21.19 ± 1.08	15.24 ± 2.16	< 0.001
Person–organization fit	15.19 ± 1.92	11.57 ± 1.58	< 0.001

## Discussion

Shortages in nursing workforce and increasing workloads have been identified as contributing factors to work-related alienation among clinical nurses, a condition in which professional roles are perceived as insufficient in meeting personal needs or professional expectations (11, 12). This condition has been associated with diminished work autonomy and reduced job satisfaction, leading to compromised quality of care and elevated turnover rates, thereby intensifying existing constraints on nursing resources (13, 14). Critical care nurses, who are required to uphold high standards of care while frequently managing patients with life-threatening conditions, are subjected to ongoing psychological stress and emotional fatigue, placing them at elevated risk for developing work-related alienation (15, 16). Previous research has shown that improvements in self-efficacy can mitigate work-related alienation by reinforcing professional competencies, affirming the value of nursing contributions, and supporting the achievement of professional goals (17, 18).

From this evidence, a checklist-based core competency training and evaluation system was developed and implemented. This approach enhances theoretical knowledge and practical skills among critical care nurses, increases training satisfaction, and significantly reduces work-related alienation. Additionally, improvements were observed in perceived organizational support and person–organization fit.

Post-intervention analysis demonstrated improvements in training satisfaction across both phases; however, significantly greater gains were observed in the post-intervention group. Furthermore, theoretical and practical examination scores were higher in the post-intervention group, indicating that the checklist-based system contributed to enhanced training outcomes and professional competency. The system functions by translating abstract job expectations into concrete, observable, and measurable behavioral indicators. Through structured guidance, the checklist provides a defined learning trajectory and self-assessment framework. This method reduces ambiguity in the learning process, enabling participants to identify and address individual gaps in competency more efficiently, thereby supporting systematic improvement in core professional capabilities.

Checklist-based formative assessment transforms traditional hierarchical evaluation into a continuous nurse-driven process of self-improvement (19, 20). Instead of functioning solely as passive recipients of evaluation, nurses engage as active participants by using checklist frameworks to plan learning activities, participate in assessment procedures, and monitor their own progress. This transformation of the learner role enhances autonomy and self-regulation, aligning with principles of intrinsic motivation. As a result, training engagement and satisfaction are elevated, self-directed learning capabilities are strengthened, and examination performance has been shown to improve (21, 22).

Post- intervention scores for work-related alienation, perceived organizational support, and person-organization fit improved in both phases, with significantly greater improvements in post-intervention compared with pre-intervention. These findings indicate that a checklist-based core competency training and evaluation system is effective in reducing work-related alienation among clinical nurses. Enhanced core competencies were identified as a foundational mechanism underlying this improvement. The critical care environment presents a complex and rapidly evolving clinical context that requires advanced clinical judgment and technical proficiency. Inadequate competency in managing these challenges often leads to feelings of helplessness and frustration, which are key contributors to work-related alienation.

The checklist system developed in this study comprehensively addressed domains including theoretical knowledge, clinical skills, communication, and applied competencies. Through structured training and systematic evaluation, substantial improvements in clinical competency were achieved. When nurses recognize their ability to perform required tasks and understand the appropriate application of their skills, professional confidence and self-efficacy are strengthened. This reduces the sense of helplessness and work-related alienation associated with competency gaps.

Perceived organizational support plays a key role in reducing work-related alienation (23). Although individual competency limitations contribute to this condition, organizational factors are equally influential. The implementation of the checklist-based system served as a visible demonstration of institutional commitment to professional development in nursing. By dedicating resources to an evidence-based training approach and prioritizing sustained professional growth, the organization conveyed support and recognition that were perceived as meaningful by the nursing staff. This perception meets emotional needs, reinforces a sense of organizational belonging and trust, and thereby reduces work-related alienation.

Improved person–organization fit represents a key outcome of the training. Alignment between individual competencies and organizational expectations fosters a higher degree of congruence. The checklist system was developed based on clinical needs and was closely integrated with professional development pathways in nursing, ensuring consistency between training content and institutional goals. The competencies cultivated through this system corresponded directly to hospital priorities, enabling nurses to perceive the realization of personal value within the organization. This alignment enhanced perceptions of meaningful work and contributed to a reduction in feelings of meaninglessness and isolation.

### Implications for nursing management

Based on the findings of this study, the checklist-based competency cultivation and evaluation system demonstrates strong potential for clinical application and broader implementation. Hospital administrators may consider incorporating this system into pre-employment training programs for new nurses and establishing it as a core tool for ongoing professional development among in-service nurses. Successful implementation depends on two key forms of support. First, resource support, which includes forming expert panels to adapt and localize the checklist, and providing standardized training for head nurses on checklist utilization and evaluation methods. Second, leadership support, whereby nursing management departments elevate the system to a strategic level within quality and safety management, fostering an organizational culture that encourages continuous learning and self-improvement. Integrating the checklist approach with routine quality control and performance evaluation processes may further ensure its sustainability and long-term effectiveness.

### Limitations and future directions

This study has several limitations. First, it employed a quasi-experimental pre–post self-controlled design, which, although practical for implementation, cannot fully eliminate potential confounding effects from time-related factors, external circumstances (such as the COVID-19 pandemic during 2020–2021), or the natural professional growth of nurses over time. Future research utilizing a randomized controlled trial design would provide stronger evidence for causal inference. Second, this was a single-center study with a relatively homogeneous sample, which may limit the generalizability of the findings. To enhance external validity, future studies should conduct multicenter investigations across hospitals of different levels and regions.

Lastly, while this study employed paired *t*-tests to compare pre- and post-intervention outcomes, repeated-measures ANOVA or mixed-effects modeling would provide a more robust approach by accounting for within-subject correlations over time. In addition, effect sizes (e.g., Cohen's *d*) were not calculated, which may limit the interpretation of the practical significance of the findings. Future studies with larger sample sizes and prospective designs are needed to incorporate these statistical approaches.

## Conclusions

In conclusion, the checklist-based core competency training and evaluation system was an effective training strategy. Through the systematic enhancement of professional competencies among critical care nurses, along with increased autonomy in training, improved satisfaction, and strengthened perceptions of organizational support and person–organization fit, work-related alienation was addressed across multiple dimensions. This approach offers nursing administrators both a theoretical basis and practical guidance for refining professional development models and promoting stability within the nursing workforce.

## Data Availability

The original contributions presented in the study are included in the article/Supplementary material, further inquiries can be directed to the corresponding author.
