# Supplementary material for: Effect of a checklist-based core competency training and evaluation system on work-related alienation among critical care nurses
Source: Front Public Health. 2026 Mar 18;14:1708734. doi: 10.3389/fpubh.2026.1708734 (PMC13038977; doi:10.3389/fpubh.2026.1708734)
Supplement: Supplementary file 1 [file Supplementary_Table_1.docx]

Supplementary Table 1. Cronbach’s α values for each scale

| Variable | Dimensions | Number of items | Cronbach’s | Reliability in Original studies |
| --- | --- | --- | --- | --- |
| Work-related alienation | Powerlessness, Meaninglessness, Self-alienation, Social alienation | 20 | 0.953 | Reliability of each dimension ranged from 0.814 to 0.893 |
| Person–Organization Fit Scale | Value congruence, Needs–Supplies fit, Demands–Abilities fit | 11 | 0.831 | 0.734–0.852 |
| Training satisfaction | Satisfaction with colleagues and personal recognition, Workload intensity, Professional development, Management policies | 10 | 0.945 | 0.942-0.952 |
| Perceived Organizational Support Scale for Nurses | Value recognition, Caring for well-being, Work support | 16 | 0.887 | 0.852–0.931 |
| McCloskey/Mueller Satisfaction Scale | 8 | 31 | 0.89 | Internal consistency coefficient = 0.97 |
